# Effect of pretreatment clinical factors on overall survival in glioblastoma multiforme: a Surveillance Epidemiology and End Results (SEER) population analysis

**DOI:** 10.1186/1477-7819-10-75

**Published:** 2012-05-03

**Authors:** Sudheer R Thumma, Robert K Fairbanks, Wayne T Lamoreaux, Alexander R Mackay, John J Demakas, Barton S Cooke, Ameer L Elaimy, Peter W Hanson, Christopher M Lee

**Affiliations:** 1 The Gamma Knife of Spokane, Spokane, WA, USA; 2 Cancer Care Northwest, Spokane, WA, USA; 3 Mackay & Meyer MDs, Spokane, WA, USA; 4 Spokane Brain & Spine, Spokane, WA, USA; 5 Gamma Knife of Spokane and Cancer Care Northwest, Deaconess Health and Education Building, 910 West 5th Ave, Suite 102, Spokane, WA, 99204, USA

**Keywords:** Glioblastoma, Asian ethnicity, Radiation, Age, Surgery, Radiation, Survival

## Abstract

**Background:**

Glioblastoma Multiforme (GBM) is one of the most aggressive primary brain tumors and is associated with a dismal prognosis. The median survival after the primary diagnosis remains poor, even after multimodal treatment approaches. However, a few patients have been reported to have long term survival greater than three years. A number of studies have attempted to define factors capable of predicting long term outcomes in specific patient groups. This article reports the outcomes of a very large group of patients diagnosed with GBM, and analyzes specific prognostic factors known to influence survival in these patients.

**Methods:**

We used the Surveillance, Epidemiology, and End Results (SEER) database of the US National Cancer Institute (NCI) to investigate various patient-related and treatment- related factors that could influence the long term survival in patients diagnosed with glioblastoma. A total of 34,664 patients aged 20 years or older with a diagnosis of GBM during the years 1973 to 2008 were studied. Overall survival outcomes were examined with Kaplan-Meier survival analysis and Cox hazard models.

**Results:**

Asian/Pacific Islanders had a better survival compared to the white population (*P* = <0.001). Patients diagnosed with GBM during the years 2000 to 2008 had a superior survival rate when compared with earlier decades (*P* = <0 .001). Statistically significant improvements in overall survival were also found for patients who received surgical resections, and adjuvant radiation treatment versus no radiation (*P*-values <0.001). Young age was also found to be highly predictive of improved overall survival rates when separated into age groups as well as when studied as a continuous variable.

**Conclusions:**

Clinical pretreatment and treatment factors, including young age at diagnosis, Asian/Pacific Islander ethnicity, recent year of diagnosis, surgical resection and the use of adjuvant radiation therapy favorably influence survival in patients diagnosed with glioblastoma.

**Trial Registration:**

All data were obtained from the United States Surveillance Epidemiology and End Results (SEER) database.

## Background

Glioblastoma remains one of the most aggressive and lethal forms of primary malignancy of the brain. However, recent advances in the diagnostics and treatments of glioblastoma have offered new hope for patients and clinicians. Despite these advances in therapy, the management of glioblastoma remains a challenge. Tumor factors such as deep infiltration of cancer cells with microscopic extensions into surrounding normal brain tissue, geographic location within the brain and a particular tumor’s accessibility to surgical resection may present barriers to adequate surgical treatment. The blood–brain barrier’s natural protection by filtration of large chemicals also limits the penetration of chemotherapeutic agents. Upon initial diagnosis, the standard treatment consists of surgery with maximal feasible resection, post-operative fractionated external beam radiation (standard dose ranges between 5,940 to 6,000 cGy in 180 to 200 cGy per fraction) with concomitant and adjuvant chemotherapy with temozolamide. The addition of temozolamide has been shown to improve overall survival. Despite this advance, the vast majority of patients still experience disease progression within a year [1].

Currently, there is not a single standard treatment for the recurrence of this tumor, although additional surgery, chemotherapy and radiotherapy are used in specific clinical circumstances. The types of salvage therapy are tailored to each patient’s clinical situation. For example, stereotactic radiosurgery (SRS) and novel chemotherapy agents are increasingly used to treat the recurrent tumors. Because of these treatment advances, outcomes have changed dramatically for appropriately selected patients. Previous studies have revealed that pretreatment prognostic factors play a role in clinical outcomes [2,3]. Several small studies have identified clinical, tumor and treatment related prognostic factors that influence outcomes [4,5]. The clinical factors of age, Karnofsky performance status (KPS), extent of surgical resection, post-operative radiation treatment, degree of necrosis within the resection pathology, and the degree of enhancement on preoperative and postoperative MR imaging studies have been shown to influence survival [6,7]. Identifying different prognostic subgroups of patients may help tailor specific treatment regimens to improve outcomes. The purpose of this study is to better analyze important pretreatment and treatment factors in the United States population associated with improvements in overall survival for patients with glioblastoma.

## Methods

All data were acquired from the 1997 to 2008 limited use databases of the Surveillance, Epidemiology, and End Results (SEER) Program of the US National Cancer Institute (NCI), which contains data from geographically specified United States locations that span a population of approximately 30 million people. Registry data are submitted without personal identifiers; therefore, patient informed consent and ethics committee approval were not required to perform this analysis.

We examined 34,664 patients aged 20 years or older with a diagnosis of glioblastoma multiforme (GBM) between 1973 and 2008. The patients were grouped by race (White, Black, Asian/Pacific Islander, American Indian/Alaska Native, Other, Unknown), diagnosis year (1973 to 1979, 1980 to 1989, 1990 to 1999, 2000 to 2008), radiation treatment (Yes, No, Unknown), extent of surgery (Surgical Resection, No Cancer Directed Resection, Unknown) and age at diagnosis (<50, >/= 50). Patients younger than 50 years were further sub-grouped into 10-year age-bands. Chemotherapy as a prognostic variable was not included since details regarding this were not available in the SEER database. Numbers of patients in the racial groups, diagnosis year groups and age groups are shown in Table 1 and numbers of patients in the radiation treatment groups and extent of surgery groups are shown in Table 2. Similarly, the distributions of patients younger than 50 years into 10-year age-bands for these same groupings are shown in Tables 3 and 4.

**Table 1:** Numbers of patients with GBM by racial groups, diagnosis year groups, and age groups

		**Age Groups**		
		**<50**	**>/= 50**	**Total**
**Racial Groups**	**Diagnosis Year Groups**			
**White**	**1973 to 1979**	542	2,149	**2,691**
	**1980 to 1989**	677	3,587	**4,264**
	**1990 to 1999**	1,226	6,307	**7,533**
	**2000 to 2008**	2,634	14,698	**17,332**
**White Total**		**5,079**	**26,741**	**31,820**
**Black**	**1973 to 1979**	26	79	**105**
	**1980 to 1989**	59	112	**171**
	**1990 to 1999**	83	287	**370**
	**2000 to 2008**	179	701	**880**
**Black Total**		**347**	**1,179**	**1,526**
**Asian/ Pacific Islander**	**1973 to 1979**	14	24	**38**
	**1980 to 1989**	23	64	**87**
	**1990 to 1999**	80	248	**328**
	**2000 to 2008**	154	570	**724**
**Asian/Pacific Islander Total**		**271**	**906**	**1,117**
**American Indian/ Alaska Native**	**1973 to 1979**	–	–	**–**
	**1980 to 1989**	–	2	**2**
	**1990 to 1999**	6	18	**24**
	**2000 to 2008**	17	44	**61**
**American Indian/ Alaska Native Total**		**23**	**64**	**87**
**Unknown**	**1973 to 1979**	–	2	**2**
	**1980 to 1989**	2	2	**4**
	**1990 to 1999**	3	6	**9**
	**2000 to 2008**	5	19	**24**
**Unknown Total**		**10**	**29**	**39**
**Other**	**1973 to 1979**	–	–	**–**
	**1980 to 1989**	–	–	**–**
	**1990 to 1999**	–	1	**1**
	**2000 to 2008**	2	12	**14**
**Other Total**		**2**	**13**	**15**
**All Races**	**1973 to 1979**	582	2,254	**2,836**
	**1980 to 1989**	761	3,767	**4,528**
	**1990 to 1999**	1,398	6,867	**8,265**
	**2000 to 2008**	2,991	16,044	**19,035**
**All Races Total**		**5,732**	**28,932**	**34,664**

**Table 2:** Numbers of patients with GBM by radiation treatment groups, extent of surgery groups, and age groups

		**Age Groups**		
		**<50**	**> = 50**	**Total**
**Radiation Groups**	**Extent of Surgery Groups**			
**Yes**	**Surgical Resection**	3,838	14,216	**18,054**
	**No Cancer Directed Resection**	548	3,764	**4,312**
	**Unknown**	159	861	**1,020**
**Yes Total**		**4,545**	**18,841**	**23,386**
**No**	**Surgical Resection**	654	4,498	**5,152**
	**No Cancer Directed Resection**	297	3,788	**4,085**
	**Unknown**	92	845	**937**
**No Total**		**1,043**	**9,131**	**10,174**
**Unknown**	**Surgical Resection**	91	505	**596**
	**No Cancer Directed Resection**	19	195	**214**
	**Unknown**	34	260	**294**
**Unknown Total**		**144**	**960**	**1,104**
**All Radiation Groups**	**Surgical Resection**	4,583	19,219	**23,802**
	**No Cancer Directed Resection**	864	7,747	**8,611**
	**Unknown**	285	1,966	**2,251**
**All Radiation Groups Total**		**5,732**	**28,932**	**34,664**

**Table 3:** Numbers of patients with GBM aged <50 by racial groups, diagnosis year groups, and 10-year age-bands

		**10-Year Age-Bands**			
**Racial Groups**	**Diagnosis Year Groups**	**20 to 29**	**30 to 39**	**40 to 49**	**Total**
**White**	**1973 to 1979**	90	134	318	**542**
	**1980 to 1989**	86	190	401	**677**
	**1990 to 1999**	117	312	797	**1,226**
	**2000 to 2008**	231	551	1,852	**2,634**
**White Total**		**524**	**1,187**	**3,368**	**5,079**
**Black**	**1973 to 1979**	7	8	11	**26**
	**1980 to 1989**	6	18	35	**59**
	**1990 to 1999**	9	19	55	**83**
	**2000 to 2008**	20	29	130	**179**
**Black Total**		**42**	**74**	**231**	**347**
**Asian/ Pacific Islander**	**1973 to 1979**	4	3	7	**14**
	**1980 to 1989**	1	7	15	**23**
	**1990 to 1999**	10	28	42	**80**
	**2000 to 2008**	20	33	101	**154**
**Asian/Pacific Islander Total**		35	71	165	**271**
**American Indian/ Alaska Native**	**1973 to 1979**	–	–	–	**–**
	**1980 to 1989**	–	–	–	
	**1990 to 1999**		3	3	**6**
	**2000 to 2008**	2	3	12	**17**
**American Indian/ Alaska Native Total**		**2**	**6**	**15**	**23**
**Unknown**	**1973 to 1979**	–	–	–	–
	**1980 to 1989**	–	–	2	**2**
	**1990 to 1999**	1	1	1	**3**
	**2000 to 2008**	–	3	2	**5**
**Unknown Total**		**1**	**4**	**5**	**10**
**Other**	**1973 to 1979**	–	–	–	**–**
	**1980 to 1989**	–	–	–	**–**
	**1990 to 1999**	–	–	–	**–**
	**2000 to 2008**	–	–	2	**2**
**Other Total**		–	–	**2**	**2**
**All Races**	**1973 to 1979**	101	145	336	**582**
	**1980 to 1989**	93	215	453	**761**
	**1990 to 1999**	137	363	898	**1,398**
	**2000 to 2008**	273	619	2,099	**2,991**
**All Races Total**		**604**	**1,342**	**3,786**	**5,732**

**Table 4:** Numbers of patients with GBM aged <50 by radiation treatment groups, extent of surgery groups, and 10-year age-bands

			**10-Year Age-Bands**		
		**20 to 29**	**30 to 39**	**40 to 49**	**Total**
**Radiation Groups**	**Extent of Surgery Groups**				
**Yes**	**Surgical Resection**	378	911	2,549	**18,054**
	**No Cancer Directed Resection**	64	111	373	**4,312**
	**Unknown**	22	41	96	**1,020**
**Yes Total**		**464**	**1,063**	**3,018**	**23,386**
**No**	**Surgical Resection**	78	152	424	**5,152**
	**No Cancer Directed Resection**	34	64	199	**4,085**
	**Unknown**	12	26	54	**937**
**No Total**		**124**	**242**	**677**	**10,174**
**Unknown**	**Surgical Resection**	6	28	57	**596**
	**No Cancer Directed Resection**	4	3	12	**214**
	**Unknown**	6	6	22	**294**
**Unknown Total**		**16**	**37**	**91**	**1,104**
**All Radiation Groups**	**Surgical Resection**	462	1,091	3,030	**4,583**
	**No Cancer Directed Resection**	102	178	584	**864**
	**Unknown**	40	73	172	**285**
**All Radiation Groups Total**		**604**	**1,342**	**3,786**	**5,732**

Survival curves were estimated using the Kaplan-Meier method and used to compare racial groups, diagnosis year groups, radiation treatment groups, extent of surgery groups and age groups. Andersen 95% confidence intervals for the median survival time of the groups were constructed. Peto’s log-rank tests were employed to determine if there is statistical evidence of differences between the survival curves of the groups. Exact conditional maximum likelihood estimates were used to calculate the groups’ hazard ratios and Fisher 95% confidence intervals were constructed for significance testing between the groups’ hazard ratios. To look at group differences between survival rates specifically at one-year, two-years, and five-years, absolute survival rates were calculated with 95% confidence intervals using a maximum likelihood solution from an asymptotic distribution by the transformation of survivorship (l_x_). Finally, the Cox proportional hazard model was used in a multivariate analysis of the racial, diagnosis year, radiation treatment, and extent of surgery categorical variables as well age as a continuous variable. Statistical significance was set at a p value <0.05. All statistical analyses utilized Stats Direct Version 2.5.7 (Stats Direct Ltd., Altrincham, UK) and Sigma Plot Version 11.0 (SYSTAT Software, Inc., San Jose, CA, USA).

## Results

**Figure 1:**
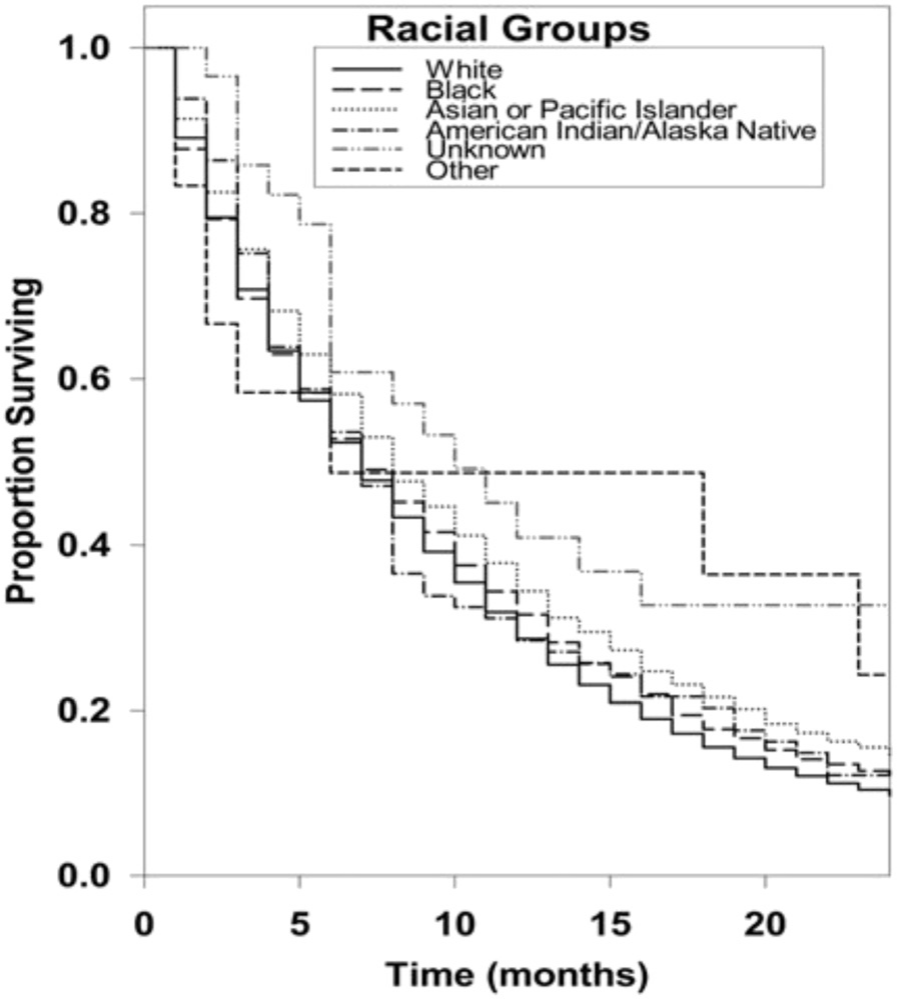
Survival curves of patients with GBM by racial group.

**Figure 2:**
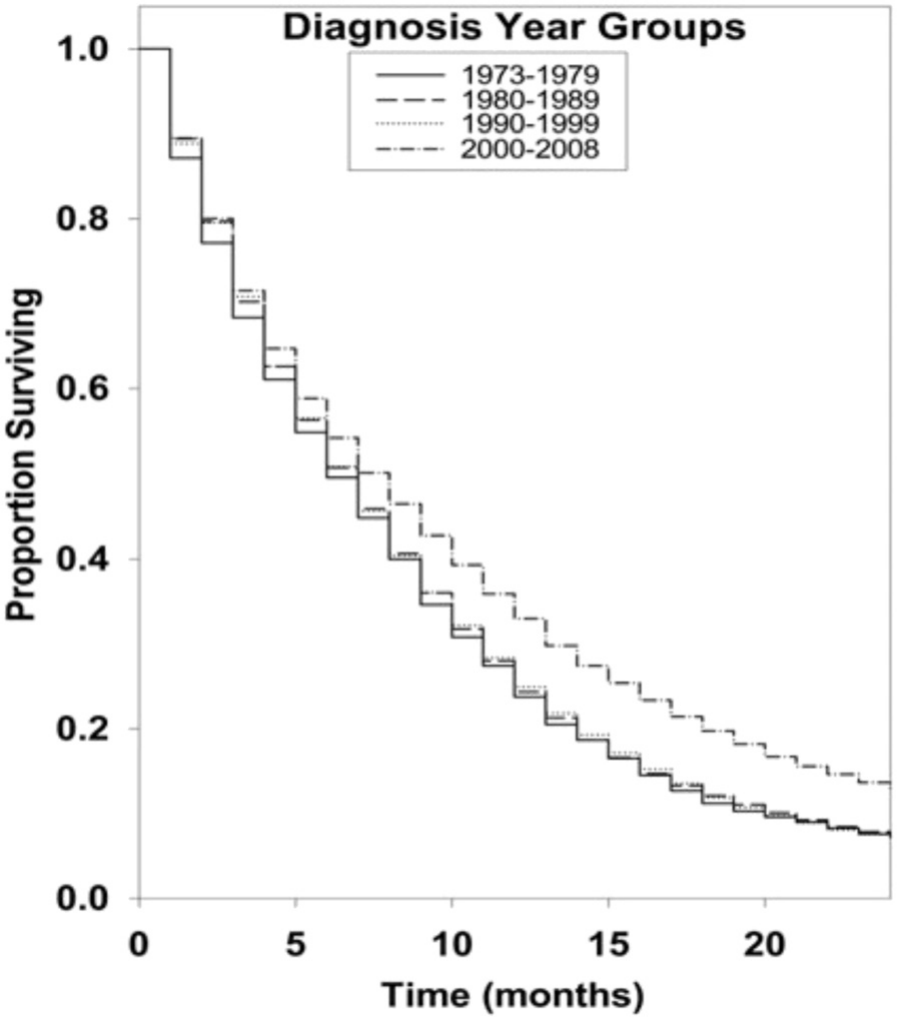
Survival curves of patients with GBM by diagnosis year groups.

**Figure 3:**
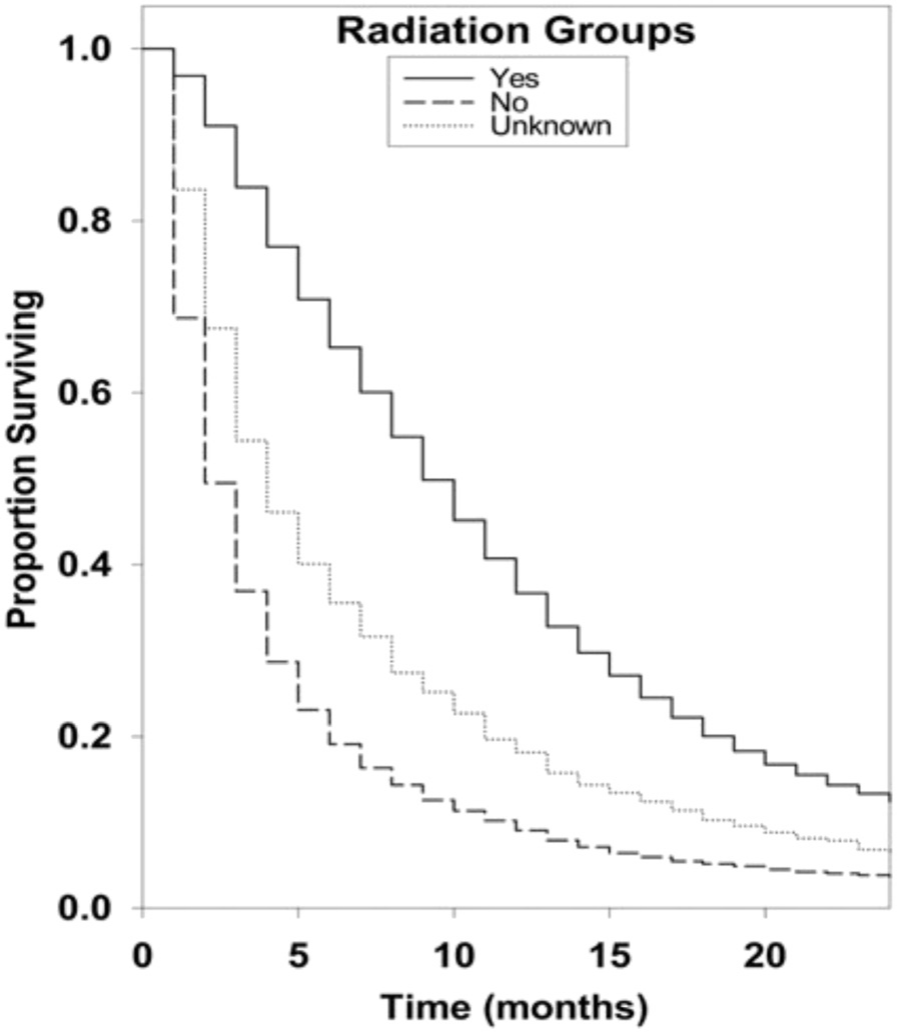
Survival curves of patients with GBM by radiation treatment groups.

**Figure 4:**
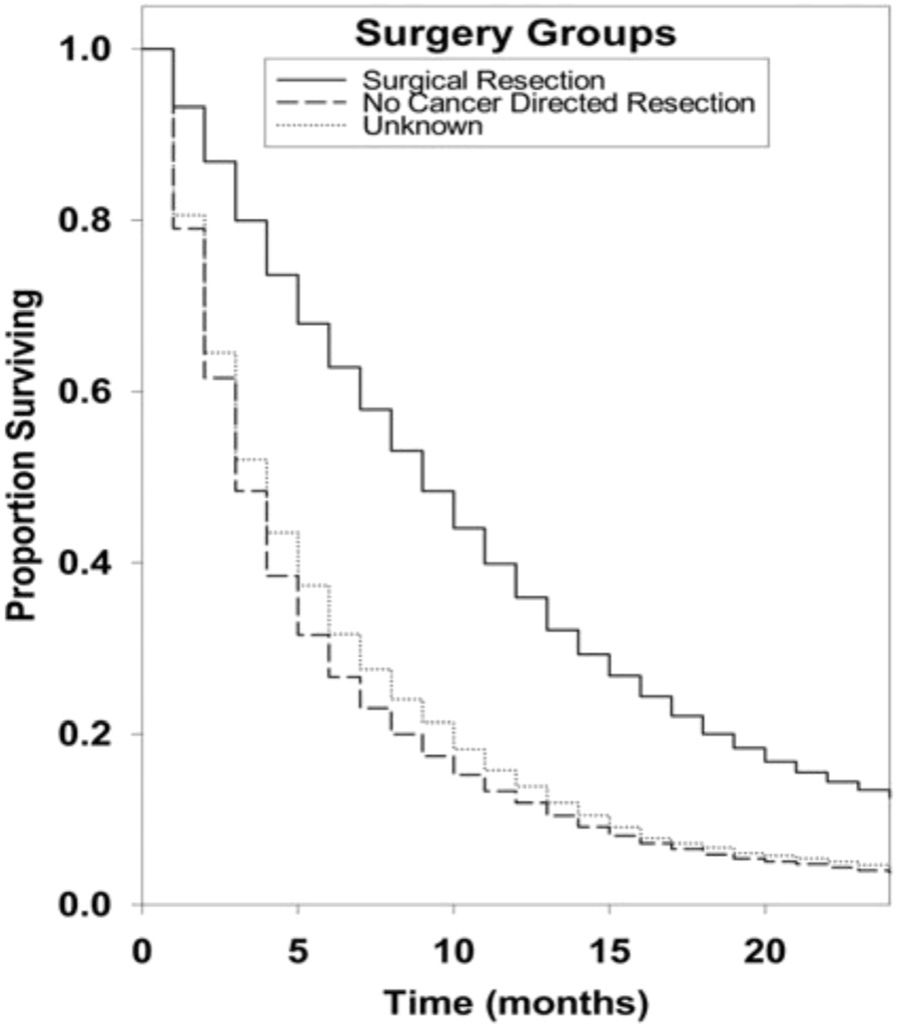
Survival curves of patients with GBM by extent of surgery groups.

**Figure 5:**
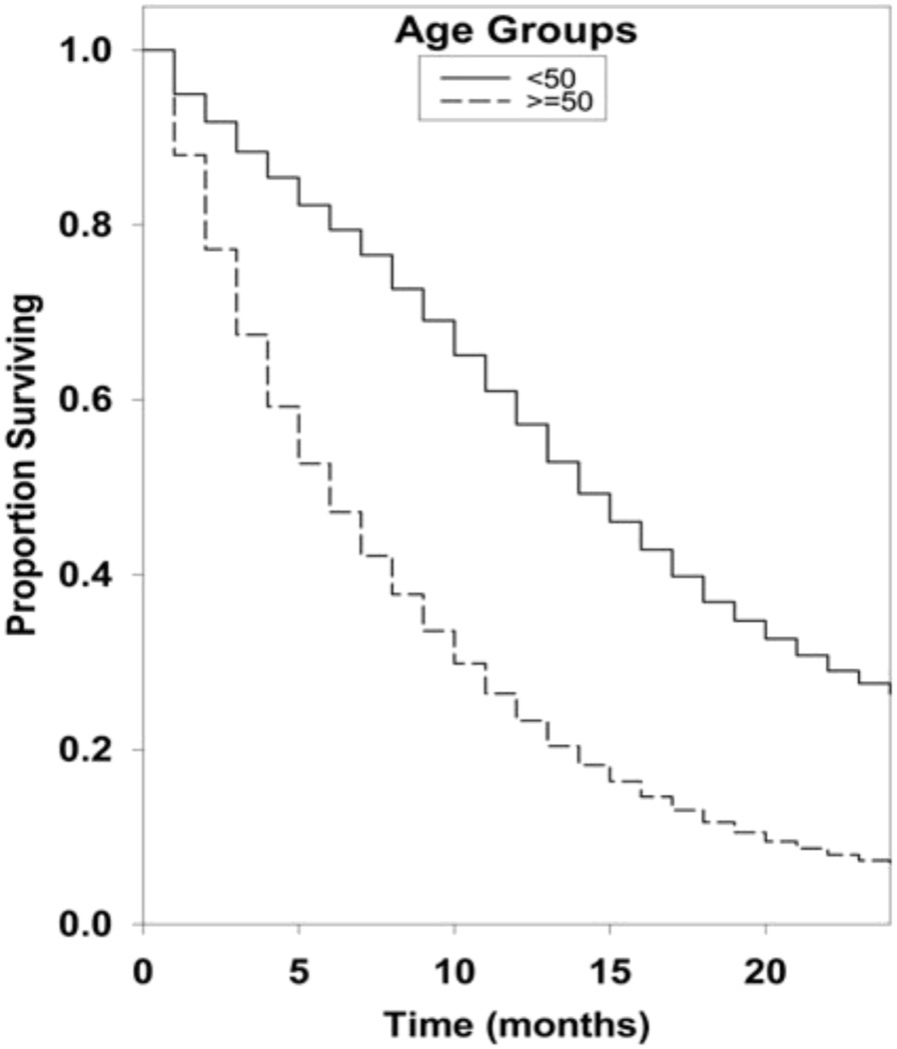
Survival curves of patients with GBM by age groups.

**Figure 6:**
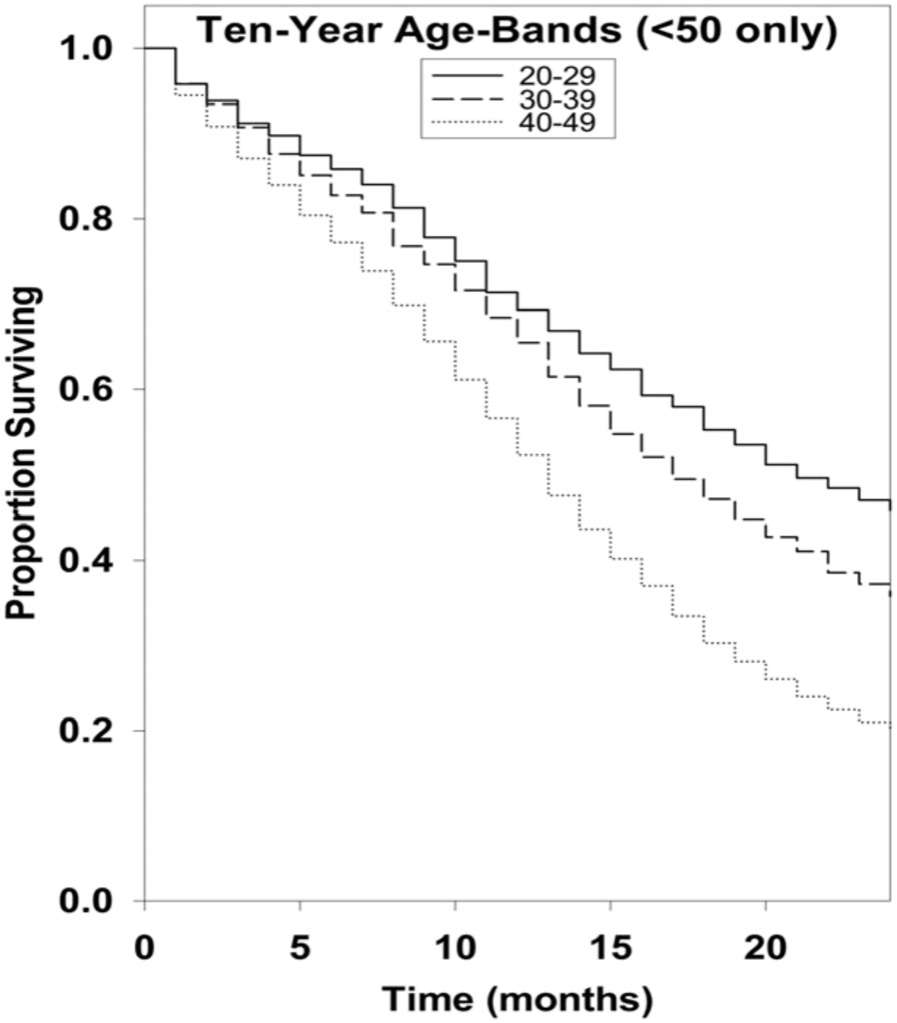
Survival curves of patients with GBM < 50 years old by 10-year age bands.

Kaplan-Meier survival curves for racial groups are depicted in Figure 1. There was a significant difference in survival of the Asian/Pacific Islander group as compared to the White group of patients. There was also evidence of significantly improved survival for patients diagnosed in 2000 to 2008 as compared to earlier years as depicted in Figure 2. Surgical resection and the use of adjuvant radiation treatment were associated with significantly improved survival outcomes as shown in Figures 3 and 4. Likewise, Figure 4 depicts improved survival for patients undergoing surgical resection. Figures 5 and 6 show significantly improved survival for patients diagnosed at younger ages.

Univariate median survival confidence interval and hazard ratio confidence intervals are included in Table 5. For each category, a reference group was selected (Race = White, Diagnosis year = 1973 to 1979, Radiation treatment = Yes, Extent of Surgery = surgical resection, Age =/<50 years, Ten-year age-band = 20 to 29 years) against which the other group’s hazard ratios were tested. The hazard ratios of the Asian/Pacific Islander and Other racial groups were found to be statistically superior to the White group (*P* < 0.001 and *P* = 0.009 respectively). All three of the later diagnosis year groups’ hazard ratios were found to be improved as compared to the reference group of 1973 to 1979 (*P* = 0.005 for both the 1980 to 1989 and 1990 to 1999 groups, *P* < 0.001 for the 2000 to 2008 group). Both the “No radiation” and “Unknown radiation” hazard ratios were determined to be inferior as compared to the “Yes radiation” reference group (*P* < 0.001 in both cases). The patients in the “No Cancer Directed Resection” group were found to have less favorable outcomes as compared to the Surgical Resection reference group (*P* < 0.001). Finally, all three variations of reviewing the impact of age indicated that patients diagnosed at older ages had less favorable hazard experiences (*P* < 0.001). Univariate 1-, 2-, and 5-year absolute survival rates for groups were also examined and are shown in Table 6. Significance was declared for a value < 0.025. Survival rate comparisons yielded broadly similar results as the univariate hazard ratio tests.

**Table 5:** Univariate median survival estimates (months) and hazard ratios of patients with GBM by racial groups, diagnosis year groups, and age groups

	**Median Survival**		**Hazard Ratio**		
	**n**	**95% CI**	**Estimate**	**95% CI**	*P*-value**
**Racial Groups**					
White*	31,820	6 ± 0.11	reference		
Black	1,526	6 ± 0.61	0.95	0.89 to 1.00	0.066
Asian/Pacific Islander	1,177	8 ± 0.67	0.83	0.78 to 0.89	<0.001
American Indian/ Alaskan Native	87	6 ± 2.03	0.86	0.68 to 1.10	0.237
Unknown	39	9 ± 6.10	0.59	0.37 to 0.88	0.009
Other	15	6 ± 19.91	0.75	0.35 to 1.43	0.467
**Diagnosis Year Groups**					
1973 to 1979*	2,836	5 ± 0.35	reference		
1980 to 1989	4,528	6 ± 0.28	0.93	0.88 to 0.98	0.005
1990 to 1999	8,265	6 ± 0.20	0.93	0.89 to 0.98	0.005
2000 to 2008	19,035	7 ± 0.18	0.78	0.74 to 0.81	<0.001
**Radiation Treatment Groups**					
Yes*	23,386	9 ± 0.13	reference		
No	10,174	2 ± 0.06	3.45	3.33 to 3.45	<0.001
Unknown	1,104	3 ± 0.26	2.50	2.33 to 2.70	<0.001
**Extent of Surgery Groups**					
Surgical Resection*	23,802	9 ± 0.14	reference		
No Cancer Directed Resection	8,611	3 ± 0.09	2.38	2.33 to 2.44	<0.001
Unknown	2,251	3 ± 0.18	0.96	0.93 to 1.00	0.126
**Age (Continuous Variable)**					
Age	34,664		1.037	1.036 to 1.038	<0.001
**Age Groups**					
<50*	5,732	14 ± 0.37	reference		
>=50	28,932	5 ± 0.10	2.22	2.17 to 2.33	<0.001
**Ten-Year Age-Bands (<50 only)**					
20 to 29*	604	20 ± 2.37	reference		
30 to 39	1,342	16 ± 1.05	1.23	1.10 to 1.39	<0.001
40 to 49	3,786	12 ± 0.39	1.85	1.67 to 2.04	<0.001

* Reference group against which other groups’ survival experience are compared.

** *P*-value for test if groups’ survival experience is same as reference groups.

**Table 6:** Univariate 1-, 2-, and 5-year absolute survival rates of patients with GBM by racial groups, diagnosis year groups, radiation treatment groups, extent of surgery groups, and age groups

	**1-year**	**95% CI**		**2-year**	**95% CI**		**5-year**	**95% CI**	
**Racial Groups**									
White*	30.54	30.03–31.05		10.11	9.76–10.46		3.25	3.02–3.48	
Black	32.42	30.06–34.80		12.10	10.44–13.88		3.31	2.34–4.55	
Asian/Pacific Islander	36.79	33.99–39.59	**	15.17	13.04–17.44	**	5.30	3.89–7.02	**
Am. Indian/Alaskan	29.41	20.16–39.26		11.51	5.71–19.54		8.12	3.22–15.97	
Unknown	47.06	29.83–62.52		32.20	15.30–50.09	**	18.40	4.95–38.54	**
Other	53.85	24.77–75.99		29.91	6.78–58.20		insufficient data		
**Diagnosis Year Groups**									
1973 to 1979*	24.79	23.21–26.39		6.86	5.97–7.83		2.30	1.79–2.90	
1980 to 1989	26.39	25.12–27.69		7.36	6.62–8.14		2.41	1.99–2.89	
1990 to 1999	26.77	25.82–27.73		7.11	6.56–7.67		2.18	1.88–2.52	
2000 to 2008	34.75	34.06–35.44	**	13.45	12.93–13.98	**	4.37	3.99–4.77	**
**Radiation Treatment Groups**									
Yes*	41.12	40.48–41.75		13.68	13.22–14.15		4.13	3.83–4.44	
No	9.25	8.70–9.83	**	3.50	3.13–3.89	**	1.74	1.46–2.05	**
Unknown	15.69	13.60–17.91	**	5.43	4.15–6.95	**	2.10	1.28–3.26	**
**Extent of Surgery** **Groups**									
Surgical Resection*	39.21	38.58–39.84		13.39	12.93–13.85		4.23	3.94–4.55	
No Cancer Directed Resection	12.78	12.08–13.50	**	3.92	3.49–4.37	**	1.44	1.16–1.76	**
Unknown	12.83	11.48–14.24	**	3.78	3.05–4.63	**	1.35	0.93–1.90	**
**Age Groups**									
<50*	58.32	57.01–59.60		26.56	25.37–27.76		11.12	10.22–12.05	
> = 50	25.46	24.95–25.97	**	7.14	6.83–7.46	**	1.67	1.49–1.87	**
**Ten-Year Age-Bands** **(<50 only)**									
20 to 29*	68.17	64.24–71.77		44.90	40.74–48.96		23.09	19.48–26.89	
30 to 39	65.31	62.66–67.83		35.55	32.89–38.21	**	16.73	14.60–18.98	**
40 to 49	54.26	52.63–55.85	**	20.38	19.05–21.75	**	7.09	6.19–8.07	**

* Reference group against which other groups’ survival experience are compared.

** Statistically significant result (*P* < 0.025) comparing whether groups’ survival rate is the same as reference group.

The multivariate analysis hazard ratio estimates and confidence intervals are included in Table 7. The multivariate analysis utilized the same reference groups as the univariate analyses against which the other group’s hazard ratios were tested. Similar to the univariate analysis, the Asian/Pacific Islander and Other racial group’s hazard ratios were significantly better than the reference group of White patients (*P* < 0.001 and *P* = 0.002, respectively). Likewise, we found similar results regarding the diagnosis year groups in that, all three later diagnosis groupings (1980 to 1989, 1990 to 1999, 2000 to 2008) had significantly superior survival as compared to the reference 1973 to 1979 group (*P* < 0.001 in all three cases). In both the radiation and extent of surgery categorical variables, the multivariate analysis found significantly better outcomes for the reference group compared to others (*P* < 0.001 in all cases). In the multivariate analysis age was also examined as a continuous variable and was found to significantly impact overall survival (*P* < 0.001) with an estimated hazard ratio of 1.037 (95% CI 1.036 to 1.038). Thus, younger age was associated with an improved survival rate.

**Table 7:** **Multivariate hazard ratios, confidence intervals, and*P***-**values of patients with GBM by racial groups, diagnosis year groups, and age**

	**Hazard Ratio**		
	**Estimate**	**95% CI**	P value**
**Racial Groups**			
White*	reference		
Black	0.97	0.91 to 1.02	0.238
Asian/Pacific Islander	0.89	0.83 to 0.95	<0.001
American Indian/Alaska Native	0.87	0.69 to 1.10	0.230
Unknown	0.51	0.33 to 0.78	0.002
Other	0.91	0.49 to 1.69	0.764
**Diagnosis Year Groups**			
1973 to 1979*	reference		
1980 to 1989	0.88	0.87 to 0.89	<0.001
1990 to 1999	0.80	0.78 to 0.81	<0.001
2000 to 2008	0.65	0.62 to 0.68	<0.001
**Radiation Treatment Groups**			
Yes*	reference		
No	2.00	1.95 to 2.06	<0.001
Unknown	1.59	1.48 to 1.70	<0.001
**Extent of Surgery Groups**			
Surgical Resection*	reference		
No Cancer Directed Resection	1.64	1.59 to 1.69	<0.001
Unknown	1.27	1.20 to 1.34	<0.001
**Age (Continuous Variable)**			
Age	1.031	1.030 to 1.032	<0.001

* Reference group against which other groups’ survival experience are compared.

## Discussion

The median survival after the primary diagnosis in patients with GBM is 12 to 16 months [8]. The patients who survive more than three years after the diagnosis are described as long term survivors [9]. It is not clear as to why a small subgroup of patients have significantly better outcomes and this could be related to clinical-, tumor- or treatment-related factors or other unknown variables. We found that the clinical factors of age less than 50 years, Asian race, surgical resection of the tumor, adjuvant radiation treatment and recent diagnosis year from 2000 to 2008 correlated with improved survival.

Most authors agree that young age at presentation is a predictor of long term survival in patients with glioblastoma [3,10]. In our study, when compared to the “<50 years” group, the “>50 years” group showed a significant decrease in survival (hazard ratio 2.22; 95% CI, 2.17 to 2.33; *P* = <0.001). When compared to “20 to 29” group, the “30 to 39 year” group (hazard ratio 1.23; 95% CI, 1.10 to 1.39; *P* = <0.001) and “40 to 49 year” group (hazard ratio 1.85; 95% CI, 1.67 to 2.04; *P* = <0.001) showed statistically significant decreased survival. Also, for every year increase in patient age, there was a significant decrease in survival (hazard ratio 1.037; 95% CI, 1.036 to 1.038; *P* = <0.001), which is a unique finding in our study. Our study results are consistent with several studies done in the past. In 1993, Chandler *et al.* estimated a mean age of 39.2 years in a group of 22 long term survivors with a median duration of survival of 9.4 years [11]. Scott *et al*. reported that 2.2% (n = 15) of a series of 689 glioblastoma patients survived more than three years, and had a mean age of 43.5 +/− 3.3 years [12]. Sneed *et al*. conducted a retrospective review to study the influence of age on the survival of patients with glioblastoma treated with radiotherapy and a brachytherapy boost. The univariate and multivariate analyses showed age as the most significant factor influencing survival (*P* = <.0005) and patients younger than 29.9 years had the highest probability of long term survival [13]. Studies by Ohgaki *et al*. reported that patients diagnosed with secondary GBM (GBMs arising from lower grade CNS tumors) survived significantly longer than those with primary GBM. However, they correlated this finding to the younger age of cases with secondary GBMs than as a reflection of different biologic behavior [14]. Many studies have emphasized the importance of age as a factor influencing survival in patients with glioblastoma, but a unique finding in our study was that for this population analysis there was a survival advantage for younger age by 10-year increments and also when examining age as a continuous variable.

Race as a factor in affecting the survival of patients with GBM has been studied in the past. The most striking finding in our study was that the Asian/Pacific Islanders had a significantly superior survival (hazard ratio 0.83; 95% CI, 0.78 to 0.79; *P* = <0.001) when compared to the White population. These data correlate with the study done by Barnholtz-Sloan *et al*. [2]. Between 1991and 1999, they studied 1,530 patients in the SEER database diagnosed with glioblastoma aged greater than 65 years of age with the intention to analyze racial differences in survival. A significant racial difference in survival was seen in the Asian population when compared to white, black and other populations. Robertson *et al*. [15] studied the incidence of glioblastoma, astrocytoma and oligodendroglioma in the white and black population in the Memphis Statistical Metropolitan area during the 1984 to 1994 period. This study did not include the Asian race, but confirmed that there were no significant differences in survival between the white and black populations despite disparity in the incidence rates. Our results confirm that racial differences in survival exist in patients diagnosed with glioblastoma, with the Asian race having increased survival when compared to other races. The reasons for this are not clearly defined. Small studies have suggested that genetic and molecular differences may play a role. There may be a higher incidence of primary glioblastomas overexpressing p 53 (Protein 53 or tumor protein 53) and PDGFR-alpha (Platelet Derived Growth Factor Receptor) similar to secondary glioblastomas in Asians [16]. However, other unknown molecular and biologic factors may play a role and this needs to be further investigated.

The management of glioblastoma has progressively changed and evolved over the course of the last two decades with new developments in technology to help with diagnosis, novel radiation techniques and advances in surgical procedures [17]. Not surprisingly, several studies have shown an improvement in outcomes over the last decade [10,18,19]. An interesting finding in our study was that the patients who were diagnosed with glioblastoma during the years 2000 to 2008 had a significantly improved survival (hazard ratio 0.78; 95% CI, 0.74 to 0.81; *P* = <0.001) when compared to other groups. The second group (1980 to 1989) with a hazard ratio of 0.93 (95% CI, 0.88 to 0.98; *P* = 0.005) and the third group (1990 to 1999) with a hazard ratio of 0.93 (95% CI, 0.89 to 0.98; *P* = 0.005) also showed improved survival when compared to the reference group. Our study shows a gradual increase in median survival in patients diagnosed with glioblastoma from the year 1973 to the year 2008. This could be related to the ever-changing patterns of care, and improvement in supportive care as well as improved radiation and surgical treatments being administered to these patients. Koshy *et al*. [19] recently reported a study involving patients diagnosed with GBM who underwent surgery and post-operative RT. These patients were selected from the Surveillance, Epidemiology and End Results database and grouped into time periods: 2000 to 2001, 2002 to 2003, 2004 and 2005 to 2006 based on year of diagnosis. They concluded that patients diagnosed in 2005 to 2006 had significantly improved survival when compared to patients diagnosed in 2000 to 2001 (HR = 0.648, 95% CI 0.604 to 0.696). This finding is consistent with our findings. With the advent of novel chemotherapeutic drugs, improved radiation techniques (including 3-D conformation radiation, intensity modulated radiotherapy (IMRT), and stereotactic radiosurgery (SRS)), advanced surgical techniques, and a better understanding of molecular biology, the median survival could be expected to increase further in the coming years. In fact, several recent studies have reported an increased number of long term survivors [20-26]. Scoccianti *et al*. studied 1,059 patients treated in 18 radiotherapy centers in Italy between 2002 and 2007 and clinical, pathological, therapeutic and survival data regarding these patients were collected and retrospectively reviewed. They reported a significant difference in survival in these patients compared to patients treated between 1997 and 2001 and attributed it to a significantly increased frequency of MRI imaging, increasing use of surgery as opposed to biopsy and use of 3-D conformal radiotherapy and temozolamide [27].

Surgical resection done after the primary diagnosis can prolong the survival, allow more comprehensive histological diagnosis and can provide relief from neurologic deficits related to the mass effect. Our study results are consistent with results of other studies [5,28]. When compared to the “Surgical Resection” group, the “No Cancer Related Resection” group showed a significantly decreased survival. This marked difference in survival emphasizes the importance of surgical resection, and also how the extent of surgical resection plays a role in prolonging the survival in patients with glioblastoma. Filippini *et al*. reported a significant difference in survival in patients who had undergone surgical resection vs patients who underwent only biopsy [29]. The hazard ratio for death in patients who had undergone surgical resection versus those who had undergone biopsy only was 0.55 (95% CI, 0.42 to 0.72; *P* = <0.001), a 45% relative reduction in the risk of death or an eight-month increase in median survival time. Gross total resection/extensive resection of the tumor at the time of initial diagnosis was associated with statistically significant increased survival when compared to sub-total resection/partial resection [29,30]. Patients older than 65 to 75 years of age, unlike younger patients, are often not offered aggressive surgery because of their age, associated comorbidities and the potential inability to tolerate surgery. In a recent study reported by Oszvald *et al*., the overall survival of older patients aged greater than 65 years (9.1 ± 11.6 months) was significantly lower than that of younger patients (14.9 ± 16.7 months; *P* = 0.0001) [31]. However, age was a negative prognostic factor in patients undergoing biopsy (4.0 ± 7.1 vs 7.9 ± 8.7 months; *P* = 0.007), but not in patients undergoing tumor resection (13.0 ± 8.5 vs 13.3 ± 14.5 months; *P* = 0.86). Survival of older patients undergoing complete tumor resection was 17.7 ± 8.1 months and compared favorably with younger patients emphasizing the importance of surgery.

Radiation therapy remains the post-operative backbone in the management of patients with glioblastoma. Our study confirms the importance of radiation treatment in prolonging the survival of patients with glioblastoma. Both the “No radiation” group (hazard ratio 3.45; 95% CI, 3.33 to 3.45; *P* =/<0.001) and the “Unknown radiation” group (hazard ratio 2.50; 95% CI, 2.33 to 2.70; *P* =/<0.001) showed a marked decreased survival when compared to the “Yes radiation” group of patients. Studies by Filippini *et al.* have shown that radiotherapy in glioblastoma patients can increase survival, with a one-third (hazard ratio 0.61; 95% CI, 0.45 to 0.83; *P* = .001) reduction in relative risk of dying [29]. Substantial research is underway to develop methods for enhancing the radio-sensitivity of GBM as most patients relapse after initial response. Tumor tissue hypoxia has been reported as an important mechanism involving tumor resistance to radiation, and using substances that can increase tumor sensitivity to radiation (radiosensitizers) is being recommended [32]. In the landmark randomized study, Stupp *et al*. reported that delivery of temozolamide during radiotherapy increased survival, suggesting that this DNA alkylating agent can increase survival by enhancing radiosensitivity of GBM cells. This study reported the overall survival rates with radiation and temozolamide to be 27.2% at two years, 16.0% at three years, 12.1% at four years and 9.8% at five years [33]. SRS is being increasingly used to treat recurrent tumors because it can target any area of the brain with extreme accuracy, thereby minimizing the effect of radiation on the adjacent brain tissue and the critical structures nearby. SRS can be used multiple times in select situations and also can be used to treat multiple sites of recurrences in the same treatment setting. Recent studies have shown that re-irradiation with stereotactic radio-surgery for recurrent glioblastoma is a very effective and feasible method of improving survival [34,35].

The complex molecular and biologic factors leading to the development of glioblastomas are beginning to be unraveled and our understanding of molecular pathogenesis has increased significantly in the last two decades. Glioblastomas are a heterogeneous group of tumors and likely arise as a result of multiple genetic alterations, including activation of oncogenes, inactivation of tumor suppressor genes or deregulation of DNA repair genes or other mechanisms [36]. Abnormal expression of tumor suppressor genes tp 53 or p 53 (tumor protein 53), PTEN (phosphatase and tensin homolog) and mdm2 (murine double minute oncogene) an important negative regulator of p53 have been implicated in the pathogenesis of GBM. Karyotyping has revealed multiple other abnormalities with significant differences between primary and secondary glioblastomas (GBM that arises as a result of transformation of lower grade gliomas). Trisomy 7, monosomy 10, allelic loss of 17p, epidermal growth factor receptor gene (EGFR) amplification are some of the other abnormalities that have been identified. TP 53 mutations are more frequent in secondary GBMs and generally do not coexist with EGFR gene amplification [37]. Secondary GBMs are associated with better outcomes compared to primary GBM [14]. Recently, other biologic factors have been reported that have been associated with favorable outcomes. Sano *et al.*[38] noted a statistically significant improved prognosis for patients with glioblastoma multiforme whose tumors expressed high levels of PTEN messenger RNA. Burton *et al*. analyzed tumors from 41 patients with GBM that survived 3 years or longer and compared them with 48 patients that survived less than 1.5 years for p53 aberrations (expression/mutation), epidermal growth factor receptor overexpression, mdm2 overexpression and proliferation index. Long-term survivors were significantly more likely to overexpress p53 (although a difference in p53 mutation rate was not observed) and significantly less likely to exhibit mdm2 overexpression, and had a significantly lower proliferation rate compared with typical GBM survivors [4]. Deletion of NFKBIA (encoding nuclear factor of κ-light polypeptide gene enhancer in B-cells inhibitor-α), an inhibitor of the EGFR-signaling pathway, promotes tumorigenesis in glioblastomas that do not have alterations of EGFR and is associated with a poor prognosis [39].

There is growing evidence that expression of O (6)-methylguanine-DNA methyltransferase (MGMT), a DNA repair enzyme that causes resistance to alkylating agents plays an important role in the pathogeneisis of glioma. Promoter methylation of MGMT leads to epigenetic silencing of the MGMT and this compromises DNA repair and has been associated with improved outcomes in patients with glioblastoma who receive alkylating agents. There is also evidence that MGMT hypermethylation and low or absent expression are frequent in oligodendroglial tumors and likely contribute to the chemosensitivity and improved outcomes of these tumors. Wiencke *et al*. reported that younger age was associated with increased incidence of TP 53 mutation and it is possible that this may be directly or indirectly related to better outcomes related to young age [40]. They also reported similar findings of increased TP 53 mutations in African Americans and Asians compared to Whites. Several studies have found an inverse relationship between glioma risk and atopy or allergy history and this is an area of ongoing research [41]. It is possible that some of these and other unknown molecular differences are associated with improved outcomes related to age, race and treatment, and future research should look into the molecular heterogeneity between different prognostic sub-groups of patients.

## Conclusions

In conclusion, to our knowledge this is the largest reported population analysis in the world literature of patient survival outcomes with glioblastoma. For a patient with glioblastoma, variables predicting longer survival include younger age (<50 years), race of the patient (Asian race being favorable), surgical excision of the tumor (gross total resection preferred), and adjuvant radiation treatment. Also, addition of temozolamide to local treatment improves survival based on randomized studies. Our study did not include assessment of chemotherapy as a prognostic variable since details regarding this were not available in the SEER database. Future research should explore the biologic differences between different prognostic subgroups of patients. With this near universally fatal disease, any small breakthrough will have a significant impact on survival and provide hope to the thousands of patients who receive this diagnosis annually. Also, the continued individualization of treatment for each unique patient’s situation will allow for improvements in survival as well as quality of life.

## Abbreviations

CNS: Central nervous system; EGFR: Epidermal growth factor receptor; GBM: Glioblastoma multiforme; IMRT: intensity modulated radiotherapy; KPS: Karnofsky performance status; mdm2: Murine double minute oncoogene; MGMT: Methylguanine-DNA methyltransferase; NCI: National Cancer Institute; p53: Protein 53 or tumor protein 53; PDGFR: Platelet Derived Growth Factor Receptor; PTEN: Phosphatase and tensin homolog; RT: radiotherapy; SEER: Surveillance, Epidemiology, and End Results; SRS: Stereotactic radiosurgery.

## Competing interests

The authors of this manuscript have no conflicts of interests, ethical conflicts or financial disclosures to make regarding this paper.

## Authors’ contributions

SRT and CML reviewed relevant literature and drafted the manuscript. BJP conducted all statistical analyses. RKF, WTL, ARM, JJD, BSC, ALE and PWH provided clinical expertise and participated in drafting the manuscript. All authors read and approved the final manuscript.
